# TB among refugees from Ukraine in European countries

**DOI:** 10.5588/ijtldopen.24.0062

**Published:** 2024-04-01

**Authors:** G. de Vries, J.P. Guthmann, B. Häcker, B. Hauer, K. Nordstrand, A. Nowinski, H. Soini

**Affiliations:** ^1^ National Institute for Public Health and the Environment, Bilthoven, The Netherlands;; ^2^ Santé publique France, Saint-Maurice, France;; ^3^ German Central Committee against Tuberculosis (DZK), Berlin,; ^4^ Robert Koch Institute, Berlin, Germany;; ^5^ Norwegian Institute of Public Health, Oslo, Norway;; ^6^ Department of Tuberculosis Epidemiology and Surveillance, National Tuberculosis and Lung Diseases Research Institute, Warsaw, Poland;; ^7^ Finnish Institute for Health and Welfare, Helsinki, Finland

**Keywords:** tuberculosis, epidemiology, refugees, screening, Ukraine

## Abstract

**BACKGROUND:**

Since the Russian Federation’s invasion of Ukraine, millions of refugees have moved to neighbouring European countries. We assessed the burden of TB in these refugees and surveyed screening approaches.

**METHODS:**

We conducted a survey among 30 European Union/European Economic Area and 13 other European countries, requesting population data on migrant residents and refugees with country of birth (COB) Ukraine, the number of TB notifications among people with COB Ukraine and countries’ screening policies for refugees from Ukraine.

**RESULTS:**

In 2021, the number of migrants born in Ukraine was 1.7 million in the 34 responding countries, and increased with 5.2 million refugees from Ukraine to 6.9 million in 2022. These countries notified 207 TB cases in people with COB Ukraine in 2021 (TB notification rate 12.0/100,000) and 887 in 2022 (TB notification rate 12.8/100,000), of which 228 (26%) had multidrug-resistant/rifampicin-resistant TB (MDR/RR-TB). TB notification rates were higher in countries advising screening for all (16.9/100,000) or specific groups of refugees from Ukraine (14.7/100,000) compared to those without screening (7.2/100,000).

**CONCLUSION:**

TB rates found in people from Ukraine were lower than the expected rate of 44 per 100,000, but higher in host countries recommending screening. Our study underscores the need for adequate TB health services for refugees from Ukraine to ensure tailored diagnosis and treatment, especially for MDR/RR-TB.

## RÉSUMÉ

**CONTEXTE:**

Suite à l'invasion de l'Ukraine par la Fédération de Russie, des millions de réfugiés se sont installés dans les pays européens voisins. Notre étude a porté sur la prévalence de la TB parmi ces réfugiés et a examiné les différentes méthodes de dépistage.

**MÉTHODES:**

Nous avons réalisé une enquête auprès de 30 pays de l'Union européenne/de l'Espace économique européen et de 13 autres pays européens, en demandant des données démographiques sur les résidents migrants et les réfugiés dont le pays de naissance (COB, pour l’anglais « *country of birth* ») est l'Ukraine, le nombre de notifications de TB chez les personnes dont le COB est l'Ukraine et les politiques de dépistage des pays pour les réfugiés d'Ukraine.

**RÉSULTATS:**

En 2021, le nombre de migrants nés en Ukraine était de 1,7 million dans les 34 (79%) pays ayant répondu à l'enquête, et a augmenté à 5,2 millions en 2022. Ces pays ont notifié 207 cas de TB chez des personnes ayant le COB Ukraine en 2021 (taux de notification de la TB 12,0/100 000) et 887 en 2022 (taux de notification de la TB 12,8/100 000), dont 228 (26%) avaient une TB multirésistante/résistante à la rifampicine (MDR/RR-TB). Les pays qui recommandent le dépistage pour tous ont enregistré des taux de notification de la TB plus élevés (16,9/100 000) tandis que ceux qui ciblent des groupes spécifiques de réfugiés ukrainiens ont signalé (14,7/100 000). En revanche, les pays ne proposant pas de dépistage ont affiché un taux de notification de seulement (7,2/100 000).

**CONCLUSION:**

Les personnes originaires d'Ukraine présentaient des taux de TB inférieurs à ceux attendus, soit 44 pour 100 000, tandis que les pays d'accueil recommandant le dépistage affichaient des taux plus élevés. Notre étude met en évidence l'importance de fournir des services de santé appropriés pour la TB aux réfugiés ukrainiens, afin d'assurer un diagnostic et un traitement adaptés, en particulier pour la MDR/RR-TB.

The full-scale invasion of Ukraine by the Russian Federation on 24 February 2022 has caused the fastest displacement crisis in Europe since the Second World War.^1^ The United Nations Refugee Agency (UNHCR) estimated that nearly one third of the Ukrainian population were forced to flee their homes. By the end of 2022, an estimated 5.9 million people were internally displaced by the war, while nearly 5.7 million refugees from Ukraine were recorded across Europe. Most have fled to Poland and Germany, but significant numbers have sought safety in or have travelled through other European countries. Ninety percent of the people leaving Ukraine are estimated to be women and children. The European Union’s Temporary Protection Directive and similar national protection schemes have provided a sound legal framework for the protection and inclusion of refugees in national systems. In 2021, the WHO estimated that TB incidence in Ukraine was 71/100,000.^2^ In 2021, Ukraine notified 19,793 TB cases, giving a notification rate of 45.5/100,000.^3^ Multidrug-resistant TB (MDR-TB) and rifampicin-resistant TB (RR-TB) was prevalent in 31% of newly diagnosed and in 45% of previously treated patients; 20% of patients are estimated to be infected with HIV.^2^

The European Centre for Disease Prevention and Control (ECDC) and WHO Regional Office for Europe recommended on 7 April 2022 not to test refugees arriving in European countries from Ukraine for TB infection or TB disease, because the TB incidence in Ukraine was less than 100 per 100,000 population.^4^ Certain groups, however, were recommended to be screened for TB disease and TB infection, such as people living with HIV, and household and other close contacts of bacteriologically confirmed pulmonary TB patients. ECDC and WHO Regional Office for Europe advise that countries applying a threshold lower than the estimated TB incidence in Ukraine should test refugees from Ukraine following their national guidelines and regulations. In this context, it is important to note that European countries have different policies and screening methods for migrants arriving in their countries.^5–8^

WHO Regional Office for Europe provided a calculator for host countries to estimate service needs for TB control, based on the assumption that the Ukrainian refugee population is composed of 65% women, 23% children and 12% men over 60 years with estimated TB incidences of respectively 50, 20 and 60/100,000,^9^ which translates into an overall TB incidence of 44/100,000. Furthermore, WHO Regional Office for Europe supported European countries in contacting the National TB Programme (NTP) of Ukraine to ensure treatment continuation for patients on treatment, e.g. in assisting transfer of consented patient data.^10^

This study aims to assess the burden of TB in refugees from Ukraine arriving in European countries and to understand its impact on the TB epidemiology in these countries. We also surveyed the different screening approaches as these also impact the epidemiology.

## METHODS

We initially conducted a survey among nine European Union (EU)/European Economic Area (EEA) countries collaborating in a network on migrant and TB issues. The network was established by participants of ECDC-supported workshops on active TB case finding implemented by the National Institute for Public Health and the Environment (RIVM), the Netherlands. The survey results were presented in a network meeting on 31 August 2023 in Copenhagen. Based on these discussions, the survey instrument was adapted and simplified, pretested in two countries, and revised accordingly, before sending it on 18 September 2023 to all TB national focal points of the 30 EU/EEA countries and 13 other European countries (Albania, Andorra, Bosnia Herzegovina, Monaco, Montenegro, North Macedonia, Republic of Moldova, San Marino, Serbia and Kosovo, Switzerland, Türkiye and the United Kingdom). Two reminders were sent to non-responding countries in October 2023.

The survey requested data on the number of migrant residents with country of birth (COB) Ukraine officially residing in countries in 2021 (available data from Eurostat 31 December 2021 was shared), the number of refugees from Ukraine with COB Ukraine end of 2022 (based on UNHCR data, if available), the number of TB notifications among people with COB Ukraine in 2021 and 2022, with additional questions on number of cases registered with continuing treatment, MDR/RR-TB and additional resistance to fluoroquinolones (pre-extensively drug-resistant TB [pre-XDR-TB]). Countries were also asked about their TB screening policies and methods for refugees from Ukraine.

For each country, we calculated: 1) The proportion of refugees from Ukraine by dividing the number of refugees on 31 December 2022 by the total country population estimated by the United Nations’ Population Division on 1 July 2022.^11^ 2) The TB rate in 2021 in people with COB Ukraine by dividing the number of notified TB cases during 2021 by the migrant resident population on 31 December 2022, both with COB Ukraine. 3) Accordingly, the TB rate in 2022 was calculated by dividing the number of notified TB cases with COB Ukraine in that year by the Ukrainian resident migrant population on 31 December 2021 *plus* the reported number of refugees from Ukraine on 31 December 2022. 4) The TB rate specifically in refugees by dividing the number of TB cases in 2022 with COB Ukraine and with arrival year 2022, by the reported number of refugees from Ukraine on 31 December 2022, as we assumed that all TB cases in 2022 with COB Ukraine and arrival year 2022 were refugees. This rate was also calculated separately for newly diagnosed cases only. Because the study only assessed anonymous data from national surveillance units, ethical approval was not required.

## RESULTS

**Figure 1.**
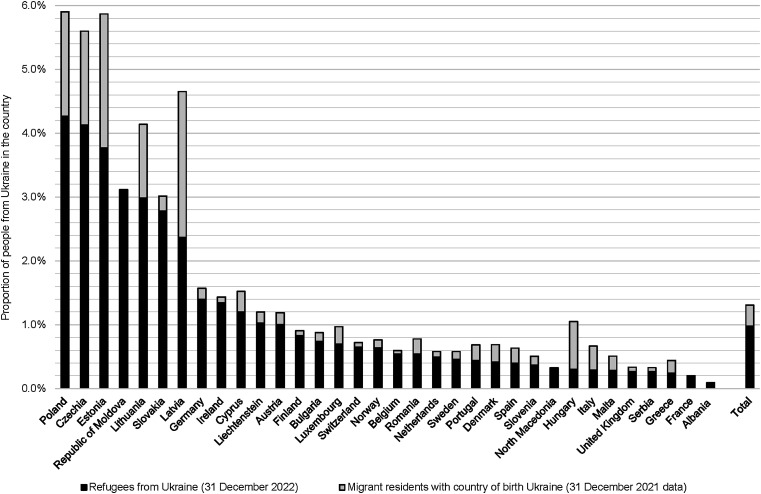
Proportion of refugees from Ukraine and migrant residents with country of birth Ukraine in responding European countries, 2022. Migrant resident data with country of birth Ukraine was not available for Albania, North Macedonia and the Republic of Moldova.

Out of the 43 contacted countries, 34 (79%) responded to the survey, 28/30 (93%) from the EU/EEA and 7/13 (54%) from non-EU/EEA countries. In 2021, 1.7 million migrant residents with COB Ukraine were registered in the responding countries; more than 600,000 in Poland and between 100,000 and 500,000 in Czechia, Germany, Italy and Spain. By the end of 2022, 5.2 million refugees from Ukraine were registered in responding countries (Table 1). Thus, the overall Ukrainian population in the responding countries increased four-fold to 6.9 million. Poland had the highest number of refugees from Ukraine (1.7 million), followed by Germany (1.2 million), Czechia (434,000), Spain (190,000) and Italy (170,000). In relation to the country’s total population, Poland, Czechia, Estonia, the Republic of Moldova and Lithuania accommodated most refugees from Ukraine in 2022 (Figure 1), amounting to 4.3% of the Polish population. The overall proportion for all responding countries was 1.0%. The 34 countries altogether notified 207 TB cases in people with COB Ukraine in 2021 and 887 in 2022, which is a 4.3-fold increase. The TB notification rate for all people with COB Ukraine in the responding European countries increased slightly from 12.0 in 2021 to 12.8/100,000 in 2022 (Table 1).

**Table 1.** Population data of European countries and migrant residents/refugees with COB Ukraine, TB notifications and incidence rates in people with COB Ukraine in 2021 and 2022, and number and proportion of MDR/RR-TB and pre-XDR-TB in 2022.*

Country name	Total country population, 2022 *n*	Migrant residents with COB Ukraine, 2021 *n*	Refugees from Ukraine, 31 December 2022 *n*	Total people with COB Ukraine, 2022 *n*	TB cases with COB Ukraine, 2021 *n*	TB rate (/100,000) in migrant residents with COB Ukraine, 2021 *n*	TB cases with COB Ukraine, 2022 *n*	TB rate (per 100,000) in people with COB Ukraine, 2022 *n*	MDR/RR-TB in people with COB Ukraine, 2022 *n* (%)	Pre-XDR-TB (proportion of MDR/RR-TB cases) in people with COB Ukraine, 2022 *n* (%)
Poland	39,857,145	651,221	1,701,600	2,352,821	68	10.4	199	8.5	51 (25.6)	5 (9.8)
Germany	83,369,843	145,510	1,164,205	1,309,715	26	17.9	282	21.5	87 (30.9)	22 (25.3)
Czechia	10,493,986	154,183	433,501	587,684	35	22.7	98	16.7	10 (10.2)	3 (30.0)
Spain	47,558,630	110,977	190,370	301,347	12	10.8	24	8.0	2 (8.3)	2 (100.0)
Italy	59,037,474	223,489	169,800	393,289	12	5.4	19	4.8	2 (10.5)	2 (100.0)
Slovakia	5,643,453	13,247	156,881	170,128	6	45.3	12	7.1	6 (50.0)	3 (50.0)
United Kingdom	56,490,048	38,030	151,000	188,030	6	16.0	10	4.8	1 (11.1)	0 (0.0)
France	64,626,628	11,407	118,994	130,401	4	35.1	33	25.3	9 (27.3)	2 (22.2)
Romania	19,659,267	45,835	106,786	152,621	0	0.0	4	2.6	0 (0.0)	0 (N/A)
Republic of Moldova	3,272,996	No data	102,000	102,000	0		26	25.5	0 (0.0)	0 (N/A)
Austria	8,939,617	16,461	89,862	106,323	0	0.0	18	16.9	5 (27.8)	0 (0.0)
Netherlands	17,564,014	15,042	86,850	101,892	0	0.0	13	12.8	4 (30.8)	2 (50.0)
Lithuania	2,750,055	31,790	82,066	113,856	2	6.3	9	7.9	1 (11.1)	1 (100.0)
Ireland	5,023,109	4,624	67,448	72,072	0	0.0	7	9.7	6 (85.7)	2 (33.3)
Belgium	11,655,930	5,673	63,398	69,071	6	105.8	12	17.4	4 (33.3)	0 (0.0)
Switzerland	8,740,472	6,175	56,623	62,798	0	0.0	3	4.8	1 (33.3)	0 (0.0)
Bulgaria	6,781,853	9,149	50,200	59,349	0	0.0	0	0.0	0 (N/A)	0 (N/A)
Estonia	1,326,062	27,828	50,000	77,828	6	21.6	16	20.6	7 (43.8)	3 (42.9)
Sweden	10,549,347	12,891	48,262	61,153	1	7.8	9	14.7	5 (55.6)	1 (20.0)
Finland	5,540,745	4,499	45,807	50,306	0	0.0	13	25.8	5 (38.5)	2 (40.0)
Portugal	10,270,865	25,443	44,960	70,403	8	31.4	9	12.8	1 (11.1)	0 (0.0)
Latvia	1,850,651	42,282	43,843	86,125	2	4.7	7	8.1	3 (42.9)	0 (0.0)
Norway	5,434,319	6,659	34,732	41,391	0	0.0	16	38.7	4 (25.0)	1 (25.0)
Hungary	9,967,308	74,482	29,978	104,460	8	10.7	36	34.5	11 (30.6)	2 (18.2)
Greece	10,384,971	20,690	25,050	45,740	1	4.8	2	4.4	0 (0.0)	0 (N/A)
Denmark	5,882,261	16,014	24,434	40,448	1	6.2	3	7.4	1 (33.3)	0 (0.0)
Serbia	6,664,449	3,969	17,875	21,844	0	0.0	2	9.2	0 (0.0)	0 (N/A)
Cyprus	1,251,488	4,000	15,023	19,023	2	50.0	1	5.3	0 (0.0)	0 (N/A)
North Macedonia	1,836,713	No data	6,322	6,322	0		0	0.0	0 (N/A)	0 (N/A)
Slovenia	2,119,844	2,989	7,748	10,737	0	0.0	2	18.6	1 (50.0)	1 (100.0)
Luxembourg	647,599	1,780	4,500	6,280	0	0.0	2	31.8	1 (50.0	1 (100.0)
Albania	2,842,321	No data	2,535	2,535	0	N/A	0	0.0	0 (N/A)	0 (N/A)
Malta	533,286	1,192	1,510	2,702	1	83.9	0	0.0	0 (0.0)	0 (N/A)
Liechtenstein	39,039	66	402	468	0	0.0	0	0.0	0 (N/A)	0 (N/A)
Total	528,605,888	1,727,597	5,194,565	6,922,162	207	12.0	887	12.8	228 (25.7)	55 (24.1)

* Countries sorted on the number of refugees from Ukraine, 31 December 2022. Data provided by survey respondents, except total country population data, which were obtained from the United Nations Division. North Macedonia and Serbia provided national data. Respondents from Albania, North Macedonia and the Republic of Moldova were unable to provide data on the number of people with COB Ukraine in 2021 in their countries. Respondent from the Republic of Moldova could not provide data on the number of refugees from Ukraine in 2022 and this data was obtained from https://reliefweb.int/report/world/refugees-ukraine-europe-january-2023-snapshot-european-countries-implement-temporary-protection-schemes-respond.

COB = country of birth; MDR = multidrug-resistant; RR = rifampicin-resistant; XDR = extensively drug-resistant; N/A = not applicable.

**Figure 2.**
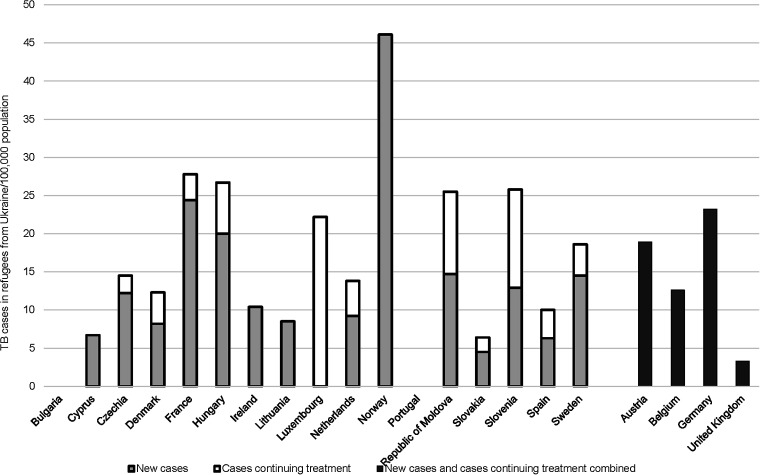
TB notification rates in 2022 in people from Ukraine who arrived in 2022 in the responding host countries, according to newly diagnosed cases and cases continuing treatment. Austria, Belgium, Germany and the United Kingdom could not differentiate TB cases into newly diagnosed and cases continuing treatment. Countries not listed reported not recording year of arrival in their national TB registers.

Twenty-one countries were able to split the number of notified cases in 2022 into cases who arrived before 2022 and in 2022 in the host country. In 2022, the notification rate for refugees with COB Ukraine in those countries was 17.4/100,000 (Table 2). Figure 2 shows that rates varied by country, with Norway having the highest rate (46.1/100,000), followed by France (27.7/100,000), Hungary (26.7/100,000), Slovenia (25.8/100,000), the Republic of Moldova (25.5/100,000) and Germany (23.2/100,000). The overall rate was 13.4/100,000 if only newly diagnosed patients were considered (Table 2, data from 17 countries).

**Table 2.** Number of TB cases and rates in people with COB Ukraine in 2022, according to year of arrival in the country and continuation of treatment (for 21 countries with available information on year of arrival).

Country name	Refugees from Ukraine, 31 December 2022	TB cases with COB Ukraine, 2022	TB cases with COB Ukraine, 2022, arrival before 2022	TB cases with COB Ukraine 2022, arrival in 2022	TB rate (/100,000) in refugees with COB Ukraine, 2022	TB cases with COB Ukraine in 2022, with arrival in 2022 and already on treatment	TB cases with COB Ukraine in 2022, with arrival in 2022 and diagnosed in the host country	TB rate (/100,000) in refugees with COB Ukraine, 2022, new cases only
	(a)	(b)	(c)	(d)=(b)-(c)	(d)/(a)	(e)	(f)=(d)-(e)	(f)/(a)
Germany	1,164,205	282	12	270	23.2	Unknown	N/A	N/A
Czechia	433,501	98	35	63	14.5	10	53	12.2
Spain	190,370	24	5	19	10.0	7	12	6.3
Slovakia	156,881	12	2	10	6.4	3	7	4.5
United Kingdom	151,000	10	5	5	3.3	Unknown	N/A	N/A
France	118,994	33	0	33	27.7	4	29	24.4
Republic of Moldova	102,000	26	0	26	25.5	11	15	14.7
Austria	89,862	18	1	17	18.9	Unknown	N/A	N/A
Netherlands	86,850	13	1	12	13.8	4	8	9.2
Lithuania	82,066	9	2	7	8.5	0	7	8.5
Ireland	67,448	7	0	7	10.4	0	7	10.4
Belgium	63,398	12	4	8	12.6	Unknown	N/A	N/A
Bulgaria	50,200	0	0	0	0.0	0	0	0.0
Sweden	48,262	9	0	9	18.6	2	7	14.5
Portugal	44,960	9	9	0	0.0	0	0	0.0
Norway	34,732	16	0	16	46.1	0	16	46.1
Hungary	29,978	36	24	8	26.7	2	6	20.0
Denmark	24,434	3	0	3	12.3	1	2	8.2
Cyprus	15,023	1	0	1	6.7	0	1	6.7
Slovenia	7,748	2	0	2	25.8	1	1	12.9
Luxembourg	4,500	2	1	1	22.2	1	0	0.0
Total	2,966,412	622	101	517	17.4	46	171	13.4

COB = country of birth; N/A = not applicable.

For 2022, the 34 responding countries reported that 228 of 887 patients (26%) had MDR/RR-TB, 226 of them were notified in EU/EEA countries. Germany (87 cases), Poland (51 cases), Hungary (11 cases) and Czechia (10 cases) observed the highest numbers of MDR/RR-TB cases (Table 1). Fifty-five (24%) MDR/RR-TB cases had pre-XDR-TB.

**Figure 3.**
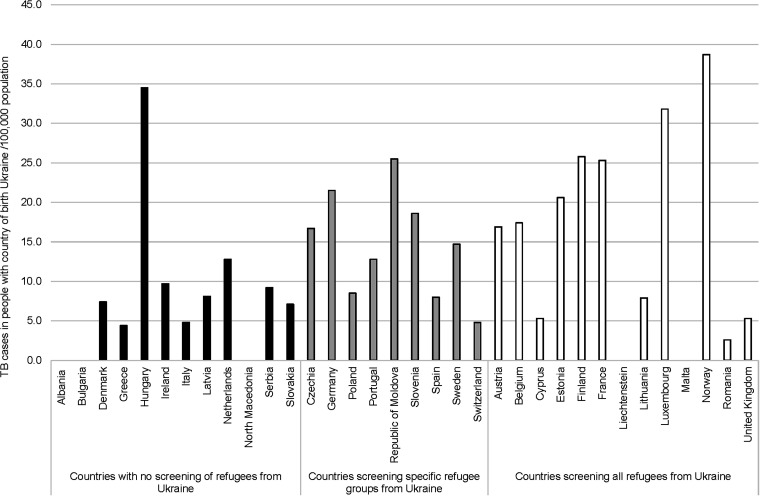
TB notification rates in people with country of birth Ukraine by national screening policy, 2022.

Thirteen countries (38%) advised to screen all refugees from Ukraine, 9 countries (26%) screened specific groups (e.g., when admitted to community facilities, before entry to kindergarten, school, work, or triaged on clinical/immunosuppressive diseases) and 12 countries (35%) were not screening refugees from Ukraine at all. In eight countries (36%) screening was mandatory and in 14 (66%) voluntary. Chest X-ray was mostly used as a screening method in 19 countries, symptom screening in 17 countries and interferon-gamma release assay (IGRA) or the tuberculin skin test in 9 countries (see Supplementary Table, multiple answers were possible). The median notification rates in countries recommending screening all refugees from Ukraine (16.9/100,000) or advising to screen specific groups (14.7/100,000) were significantly higher than for countries without screening recommendations (7.2/100,000) (*P* < 0.01 for both comparisons). The difference in rates between the two first groups was not statistically significant (*P* = 0.10) (Figure 3).

## DISCUSSION

More than 5 million refugees from Ukraine were registered at the end of 2022 in the 34 European countries responding to our survey 2022, which gave four times increase of the Ukrainian migrant population. Countries bordering Ukraine received relatively higher numbers of refugees. In the responding countries, the number of TB cases notified in people with COB Ukraine in 2022 was more than four times higher than in 2021. Consequently, TB notification rates in people with COB Ukraine increased slightly from 12.0 to 12.8/100,000 population. Considering only the refugee population in 2022, the notification rate was 17.4/100,000 for all, and 13.4/100,000 for newly diagnosed patients only; 26% of patients had MDR/RR-TB, 24% of them pre-XDR-TB.

If the WHO Regional Office for Europe calculator-based estimate of 44 cases/100,000 Ukrainian refugee population was applied to the 5.2 million refugees observed in our study, then 2,301 TB cases would have been expected among these refugees in 2022; 2.6 times higher than the total number of 887 cases notified by the countries responding to our survey. By restricting the calculations to countries who were able to provide data disaggregated by year of arrival, 1,314 cases would have been expected by WHO, which is 2.5 times higher than the 516 cases reported. There are several of explanations for the differences seen between country data and WHO estimates. First, the WHO calculation is based on an estimate of the TB incidence which is usually higher than the actual number of cases diagnosed and notified. It is of interest to see that countries advising screening observed higher notification rates, which were closer to the WHO-estimated TB incidence, with Norway even exceeding the WHO estimate. Second, refugees had stayed less than 10 months in European countries by the end of 2022, so the numbers would be higher if they had stayed a full year in the country.

The 226 MDR/RR-TB cases with COB Ukraine reported in 2022 by the responding EU/EEA countries in our study predominantly explains the 26 per cent increase of notified MDR/RR-TB cases in the EU/EEA, from 739 in 2021 to 933 in 2022.^12^ The proportions of MDR/RR- and pre-XDR/XDR-TB in our study are comparable to those reported by Ukraine in 2022. In Germany and Poland, the overall MDR/RR-TB case numbers almost doubled in 2022 compared to the previous year; in Czechia an increase to 60% was seen. This clearly shows that countries should develop contingency measures when demand on existing health services increases.^13^ Poland, in partnership with the WHO and non-governmental organization Doctors Without Borders, e.g., placed emphasis on providing outpatient treatment for drug-resistant TB through short treatment regimens and establishing a more inclusive system for migrants. Key initiatives included a Ukrainian-language helpline for TB patients, extensive information exchange with Ukraine, video support treatment for patients, as well as rapid molecular diagnosis of MDR/RR-TB.

Sixty-five per cent of European countries advised to screen all or specific groups of refugees from Ukraine, which is higher than the 51% reported in a recent survey.^14^ In our study, TB notification rates were two times higher in countries recommending screening than in countries not recommending screening. It is well-known that up to 50% of pulmonary TB is asymptomatic or subclinical and missed without radiological screening.^15^ Several countries reported that the uptake of screening is affected by the way refugees from Ukraine are accommodated and screening is organised. In Norway, screening is mandatory, and coverage is estimated to be >90% in refugees from Ukraine.^16^ In France, a survey involving TB public health clinics, found that more than half of the TB cases among refugees from Ukraine were identified through active screening; the screening yield was 116/100,000.^17^ Authors stated that screening coverage was low. In Germany, screening is mandatory for refugees from Ukraine admitted to community facilities,^18^ but only around 20% were staying in shared accommodation. Nevertheless, approximately 40% of notified TB cases with COB Ukraine were identified through screening.^18^ In a German study, the TB screening yield in refugees from Ukraine was 183/100,000; 13% of those receiving an IGRA tested positive.^19^ In Wales, national screening identified one TB case (yield: 51/100,000) and 6.5% IGRA positivity in refugees from Ukraine.^20^ In Czechia, screening measures recommended by professional societies were not applied since the influx of large number of refugees created a great strain on the Czech healthcare system.^21^ These different findings emphasise the importance of evaluating the coverage and effectiveness of TB screening.^22^ Furthermore, the epidemiological TB situation in Ukraine can deteriorate as the war continues.^23^ According to the WHO, the estimated TB incidence in Ukraine increased from 71 in 2021 to 90/100,000 in 2022.^24^ Although this is probably explained by a change in the population composition in Ukraine, it can also represent a true increased TB risk due to transmission and less access to health services and may affect TB risks in future refugees.

Thirteen countries (38%) responding in our survey were not able to provide data on year of arrival, an important variable to assess the refugee TB burden. Furthermore, we noted that countries had different procedures in notifying and recording cases already on treatment and continuing treatment in the host country. Some countries include and others exclude them as a TB case in the national data and when reporting to ECDC. We recommend that, under guidance of ECDC and WHO Regional Office for Europe, countries consider improving their TB surveillance systems to be able to identify cases in newly arrived migrants/refugees and differentiate cases that started treatment before entry in another country. We further recommend informing the national TB authorities in the country where the initial diagnosis was made (here, mostly the NTP Ukraine) of the treatment outcome. Authors´ experiences regarding communication and consented patient information exchange with the NTP Ukraine so far have been excellent and in line with recommendations on cross-border TB control.^25,26^

The strength of our study is the high response rate by national focal points, providing the best available national TB notification data, and using UNHCR and Eurostat data where available. However, it remains challenging to get the appropriate denominator population data of such mobile, sometimes transiting and therefore unstable migrant groups. The calculations are imperfect, but the best we can get. As granular denominator data were lacking, we did not ask for detailed demographic data of notified TB cases, such as sex and age, or disease-related factors, such as culture confirmation. This limits drawing conclusions on specific refugee groups.

## CONCLUSION

Our study underscores the need for adequate TB health services for refugees from Ukraine in European countries to ensure tailored diagnosis and treatment, especially for MDR/RR-TB. The TB rates found in people from Ukraine were lower than the expected rate of 44/100,000, but higher in host countries recommending TB screening. The benefits of screening are still under debate and improved collection and evaluation of screening data and yield are needed. We suggest that countries, including Ukraine, and international organisations, discuss screening of refugees from Ukraine and possibly develop a more evidence-based and coordinated approach.

## Supplementary Material


