# Supplementary material for: TB among refugees from Ukraine in European countries
Source: IJTLD Open. 2024 Apr 1;1(4):166–73. doi: 10.5588/ijtldopen.24.0062 (PMC11231824; doi:10.5588/ijtldopen.24.0062)
Supplement: Supplementary file 1 [file iutld_ijtld_open_24.0062_supplementarydata1.pdf]

©2024 The Union http://dx.doi.org/10.5588/ijtldopen.24.0062

TB among refugees from Ukraine in European countries

Supplementary Table S1: European countries' tuberculosis screening policy in refugees from Ukraine and screening methods
(De Vries *et al.* Tuberculosis among refugees from Ukraine in European countries. IJTLD-02-24-0062)

**Country name**	**Screening policy in refugees from Ukraine***	**Mandatory of voluntary screening**	**Screening method(s) mostly applied †**		
			**Symptom screening**	**Chest X-ray**	**TST/IGRA**
Albania	No				
Austria	Yes, all refugees	Mandatory		X	
Belgium	Yes, all refugees	Voluntary	X	X	
Bulgaria	No				
Cyprus	Yes, all refugees	Mandatory	X	X	X
Czechia	Only specific groups	Voluntary	X	X	
Denmark	No				
Estonia	Yes, all refugees	Voluntary		X	X
Finland	Yes, all refugees	Voluntary	X	X	
France	Yes, all refugees	Voluntary		X	
Germany	Specific groups	Mandatory		X	
Greece	No				
Hungary	No				
Ireland	No				
Italy	No				
Latvia	No				
Liechtenstein	Yes, all refugees	Voluntary	X		
Lithuania	Yes, all refugees	Mandatory	X	X	X
Luxembourg	Yes, all refugees	Mandatory	X		
Malta	Yes, all refugees	Mandatory	X		
Netherlands	No				
North Macedonia	No				
Norway	Yes, all refugees	Mandatory		X	
Poland	Only specific groups	Voluntary	X	X	
Portugal	Only specific groups	Voluntary	X	X	
Republic of Moldova	Only specific groups	Mandatory	X	X	X
Romania	Yes, all refugees	Voluntary	X	X	
Serbia	No				
Slovakia	No				
Slovenia	Only specific groups	Voluntary	X	X	
Spain	Only specific groups	Voluntary	X		X
Sweden	Only specific groups	Voluntary	X	X	X
Switzerland	Only specific groups	Voluntary	X		
United Kingdom	Yes, all refugees	Voluntary	X	X	X

Abbreviations: IGRA = Interferon Gamma Release Assay; TST = Tuberculosis Skin Test

* Definition of screening (in the survey): systematically examining all or specific groups of people for TB. People who report with symptoms and are referred for further investigation, are not considered as being diagnosed by active case finding/screening.

† More than one answer possible.
